# Cannabinoid hyperemesis syndrome presenting with ventricular bigeminy

**DOI:** 10.1186/s42238-023-00203-x

**Published:** 2023-10-19

**Authors:** Jeffrey Wong, Muneet Gill, Thor Stead, Derrick Huang, Latha Ganti

**Affiliations:** 1 University of Central Florida, Orlando, FL USA; 2 Brown University, Providence, RI USA; 3 Warren Alpert Medical School of Brown University, Providence, RI USA; 4 Ocala Regional Medical Center, Ocala, FL USA; 5 University of Central Florida College of Medicine, Orlando, FL USA

**Keywords:** Cannabinoid hyperemesis syndrome, Ventricular bigeminy, Premature ventricular contractions

## Abstract

The is a case of a 28-year-old male presenting to an emergency department (ED) via emergency medical services (EMS) with a chief complaint of “gastritis.” He was noted to have bigeminy on the pre-arrival EMS electrocardiogram. He was ultimately diagnosed with cannabinoid hyperemesis syndrome (CHS). CHS is becoming an exceedingly common emergency department presentation due to the poorly regulated but widespread availability of cannabis products. The authors discuss a case of CHS and ventricular bigeminy.

## Introduction

Cannabinoid hyperemesis syndrome, also known as CHS, is a type of cyclic vomiting syndrome that causes nausea and severe vomiting. The condition is most prevalent among young adult males (Perisetti et al. 2020). CHS typically occurs secondary to daily to weekly cannabis use. Prevalence of CHS is increasing with time as the percentage of the active compound delta-9-tetrahydrocannabinol (THC) in cannabis available legally and illicitly has been steadily increasing for the last few decades (Lapoint et al. 2018). The research supporting cannabis as an anti-emetic was done when the concentration of cannabis was less than 10% in the smoked plant. Cannabis preparation today ranges from 18 to 95% THC, on which no validated research is available to date (Simons 2021). The advent of “medical cannabis” cards which are easily obtained even by children further compounds this problem (https://www.medpagetoday.com/publichealthpolicy/publichealth/90980). Each year, 2.75 million cases of CHS are reported in the USA alone (Habboushe et al. 2018). A systematic review characterized the most common symptoms of CHS as follows: compulsive hot baths with symptom relief (92.3%), male predominance (72.9%), abdominal pain (85.1%), and at least weekly cannabis use (97.4%) (Sorensen et al. 2017). Unfortunately, many cannabis users reject the possibility that cannabis is the etiology for their cyclic vomiting or that cessation of cannabis use is an effective treatment (Simons 2021). The treatment for CHS is cessation of cannabis use. This treatment is 100% effective. The authors report on a case of CHS and ventricular bigeminy, as we are not aware of any published reports associating of cannabis use or CHS with ventricular bigeminy.

## Case presentation

The patient is a 28-year-old male who presents to the emergency department via EMS directly after coming off the airplane from a domestic flight. His chief complaint is gastritis. The patient states that he gets this gastritis quite frequently. When asked to describe his gastritis, he endorsed pain in the epigastrium, nausea, and a feeling of indigestion. He explains that he goes to an emergency room for gastritis about once a month. He says, “they never find nothing.” He explains he had an endoscopy a month ago which was negative. The patient actively smokes cannabis (for the last several years) on a daily basis tobacco and drinks alcohol. Last night, he had a “blunt” which he “eased down” with some alcohol. He says this often brings on his gastritis. When asked to describe his “gastritis,” he explained he had suffered numerous episodes of intractable vomiting that came on suddenly. He says the episodes are preceded by severe nausea and then vomiting that is cyclical and not easily relieved with usual measures. He usually tries hot baths which help briefly but then ends up in the emergency room where they give him intravenous medication. The symptoms abate entirely when he quits cannabis for several days.

**Fig. 1:**
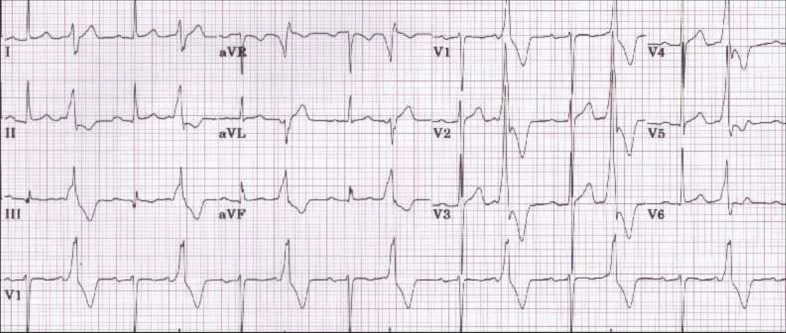
Electrocardiogram demonstrating ventricular bigeminy

When the paramedics picked him up, they noticed that he had some ventricular bigeminy on the rhythm strip. This was confirmed in the emergency department here with a 12-lead ECG (Fig. 1). The patient denies any medical history except for this “gastritis.” He denies any cardiac history. He denies any family history of heart disease. He denies taking any medication daily. His vital signs were within normal limits. His blood pressure was 121/58 mmHg, temperature 98.6 °F, respirations 16 breaths per minute, pulse 90 beats per minute, and he was saturating at 99% room air. The patient appeared uncomfortable but was not toxic and was cooperative with the physical exam. His abdomen was soft, non-tender, and nondistended, but the patient did have voluntary guarding. There was no costovertebral angle tenderness. The remainder of the physical examination was unremarkable.

 Laboratory analysis revealed leukocytosis and a urine toxicology screen positive for cannabinoids only. Blood alcohol level was negative. Computed tomography scans of the abdomen and pelvis revealed no acute pathology. Given the patient’s history and pattern of cannabis abuse and his presenting symptoms of extreme vomiting and retching with no acute pathology found, a working diagnosis of cannabis hyperemesis syndrome was made. For this, he was given 2 mg of intravenous haloperidol. He was also given 20 mg intravenous famotidine for gastritis. Patients’ nausea and retching resolved promptly. He was admitted to the hospital for a 23-h observation. His gastrointestinal symptoms resolved as did his ventricular bigeminy. He was discharged home after being counseled regarding cannabis use. On routine 72 h post-discharge follow-up, the patient had abstained from cannabis during that time and indeed he had no further episodes of vomiting. He reiterated that when he does not smoke cannabis, he does not have the vomiting. However, he did plan to go back to smoking cannabis once he would feel better. We counseled him on CHS but he said he smoked almost daily and that it would be too difficult to quit.

## Discussion

The patient’s description of his “gastritis” is typical of CHS, with severe nausea and cyclical vomiting that resolves after cannabis cessation in a person using cannabis for many years, as supported by the Sontineni and Rome IV criteria (Sontineni et al. 2009; Stanghellini et al. 2016; Venkatesan et al. 2020). Specifically, the patient’s symptoms had been present for more than 6 months and consisted of stereotypical episodic vomiting resembling cyclic vomiting syndrome in terms of onset, duration, and frequency. These occurred after prolonged use of cannabis, and when he stopped cannabis, the vomiting would subside.

Because the symptoms of CHS present closely to that of cyclic vomiting syndromes, the diagnosis can be delayed. This delay in diagnosis can also be partially attributed to the social stigma surrounding cannabis use. In this case, the patient was very upfront about their frequent cannabis and alcohol use, which was critical information in reaching this diagnosis. However, the patient’s recurrent gastritis was dismissed multiple times and not seriously considered as a possible symptom of CHS. This is likely because the typical chief complaint associated with this syndrome is nausea or vomiting. It is important to note that esophagogastroduodenoscopy findings from several patients with CHS have revealed varying grades of esophagitis and gastritis (Galli et al. 2011). As cannabis and cannabis products become more widely used, it is imperative that clinicians familiarize themselves with evidence that shows the paradoxical effects that it has on the gastrointestinal tract (Habboushe et al. 2018). Although in small doses cannabis can actually be an anti-emetic, overuse and using higher doses can result in CHS. Sometimes, patients will take more at the first sign of nausea, because of this. Knowing a patient’s drug history helps clinicians more quickly arrive at the diagnosis.

The cannabinoid receptor type 1 (CB1) receptors stimulated by cannabis are key in the pathophysiology and treatment of CHS. These receptors are present in both the central nervous system (CNS) and the enteric plexus and are responsible for cannabis’s neurologic and gastrointestinal effects (Patterson et al. 2010; Hickey et al. 2013). Although cannabis has been utilized for the treatment of nausea, chronic or significant cannabinoid use can produce the paradoxical effects of cyclic vomiting as seen in CHS (Patterson et al. 2010). One possible explanation is that CB1 receptor stimulation has been shown to slow gastric emptying and inhibit peristalsis, which may play a role in the pathogenesis of CHS (Chang and Windish 2009). Additionally, the presence of these receptors near the thermoregulatory center of the hypothalamus may explain the association of warm bathing with relief in symptoms (Patterson et al. 2010; Pierard and Hantson 2017). Typical acute treatment regimens have included intravenous fluid hydration, antiemetics, and benzodiazepines. However, when these approaches fail, the dopamine D2 receptor blocker, haloperidol, has been utilized—as in our patient. (Venkatesan et al. 2020). The effectiveness of haloperidol in CHS may be explained by the association of D2 receptors in the chemoreceptor trigger zone associated with emesis and the role of haloperidol in interfering with the known complex interactions between dopamine and CB1 signaling mechanisms (Hickey et al. 2013; Witsil and Mycyk 2017).

The patient also presents with ventricular bigeminy, which occurs when the heart alternates between a normal sinus rhythm and a premature ventricular complex (PVC). PVCs are often associated with structural heart disease and increase the risk of sudden death in people with this condition (Ahn 2013). The magnitude of the mortality risk depends on the severity of the underlying disease. Risk factors for PVCs include underlying ischemic heart disease, advanced age, male gender, hypertension, hypomagnesaemia, hypokalemia, and bundle branch block (Farzam 2021). The patient did not present with any of these risk factors. In the absence of heart disease, PVCs carry no adverse prognostic significance if the individual is under the age of 30 (considered to be benign), as seen in this case (Ahn 2013). Although there are no established links/causal relationship between ventricular bigeminy and CHS, cannabis use has been reported to trigger coronary vasospasm, which can result in ischemic ECG changes resembling acute coronary syndromes (Pierard and Hantson 2017).

A literature search of [cannabinoid hyperemesis syndrome] OR [cannabis] AND [ventricular bigeminy] OR [bigeminy] conducted in December 2021 did not identify any studies. The search covered PubMed and Embase, included years 2000–2022, and was limited to articles in English. Since then, there has been one case report that reported on the association of cannabis with myocardial infraction (Aissaoui et al. 2022) and one study that examined the association of self-reported cannabis use with cardiac arrhythmias (Harding et al. 2022). The cardiac arrhythmias noted in the latter included supraventricular tachycardias and PVCs. Premature ventricular contractions can be a precursor to ventricular bigeminy.

While it may seem counterintuitive to give haloperidol for ventricular bigeminy, a phase IV trial of 21,383 people who took haloperidol revealed that only 1 person (0.0%) experienced bigeminy (Haldol and Ventricular 2023).

How cannabis can produce ventricular bigeminy can perhaps be explained by the presence of CB1 receptors in the myocardium. Their activation may result in increased generation of reactive oxygen species that predispose vasospastic responses (Patterson et al. 2010). In addition, the well-known increased sympathetic nervous system activity from cannabis may also result in myocardial changes. These effects on the myocardium mirror the association of PVCs with structural heart disease and may be key in explaining the ventricular bigeminy seen in our patient (Patterson et al. 2010).

It is possible that ventricular bigeminy is associated with cannabis use, rather than CHS per se. To the authors’ knowledge, bigeminy is not associated with other vomiting disorders. Dehydration and electrolyte imbalances may also contribute to bigeminy, but our patient did not display any overt signs of these pathologies.

## Conclusion

The authors report a case of CHS associated with ventricular bigeminy. As the use of cannabis increases in society, CHS-related phenomena will likely be noted with greater frequency. This case highlights the widespread impact of cannabis on the cardiovascular system.

## Data Availability

All data generated or analyzed during this study are included in this published article and its supplementary information files.

## Abbreviations

CHS: Cannabinoid hyperemesis syndrome
PVC: Premature ventricular contractions
